# Jottings

**Published:** 1910-03-26

**Authors:** 


### Jottings.

The Prince and Princess of Wales have sent a gift of volumes of the *Nineteenth Century* to St. Mary's Hospital.

So far £500 has been received for the Forfar Infirmary towards the proposed extension scheme, which is expected to cost £1,000.

Mr. and Mrs. W. H. Heywood, of Holly Mount, Edgerton, have publicly presented to the Huddersfield Infirmary a fully-equipped nursing home.

In spite of the record number of in-patients received last year at the Royal Ear Hospital Sir Ernest Hatch, at the Governors' annual meeting, declared that no death had occurred.

The Princess of Wales has given her patronage to the bazaar to be held on May 24 and 25 in aid of the Hospital for Women, Soho Square, to complete by £4,500 the fund for the rebuilding of the hospital.

Princess Henry of Battenberg was present on Wednesday afternoon, the 16th, at a concert in aid of the Belgrave Hospital for Children, held at 54 Mount Street, by permission of the Countess of Plymouth.

The General Committee of the Coventry and Warwickshire Hospital has agreed to recommend the appointment of an oral specialist, as great success had attended the analogous appointment of ophthalmic surgeon.

Prince Christian presided at the recent annual meeting of the subscribers to King Edward VII. Hospital, Windsor. Last month permission was given for the two main wards to be styled King Edward's ward and Queen Alexandra's ward.

The first annual meeting of subscribers to the Lydney Cottage Hospital (Glos.) since its reconstruction was presided over by Mr. Richard Beaumont Thomas, when a balance of £133 on the right side was announced to be carried forward.

The annual festival dinner of the Royal Hospital for Incurables will be held at the Grocers' Hall, Princes Street, E.C., on Wednesday, May 11, 1910, at 6.30 p.m. for 7 p.m. precisely, Sir Horace Brooks Marshall, M.A., LL.D. (Alderman of the City of London), in the chair.

The first annual meeting, under the presidency of the Mayor, of the Paddington Dispensary has now been held. It is modelled on the Royal Victoria Dispensary for Consumption at Edinburgh, which was founded twenty years ago by Dr. R. W. Philip. Dr. Philip, Sir Malcolm Morris, Sir John Broadbent, and Dr. Dudfield also addressed the meeting.

The General Committee of Addenbrooke's Hospital, Cambridge, has resolved that a gift of £1,000 shall entitle the benefactor to have a bed named after him or her in perpetuity, and a gift or bequest of £500 to have a cot so named, and that annual subscriptions of £50 or £25 shall during the continuance of the subscription entitle the subscriber to have a bed or cot named after him or her.

The fifty-seventh annual report of the Charity Commissioners has been issued as a Parliamentary paper, and recalls the recommendations of the Poor Law Commission. Medical charities receive by far the largest share of the endowments created last year, namely, £1,237,892. Bequests to medical charities to which no trusts attached amounted to £442,000. The case of the "Attorney General *v.* Belgrave Hospital" is recorded as affirming the continual practice of the Commission in regard to gifts for beds or cots by the judgment of Mr. Justice Swinfen-Eady.

New annual subscriptions and donations amounting to £66 10s. were announced at the annual meeting of the Hospital for Epilepsy and Paralysis, Maida Vale, by governors unable to attend.

A new nurses' home is badly wanted at the Metropolitan Hospital, Kingsland Road, which would cost £7,000. If this were built the number of beds in the hospital could be raised by 36, making a total of 150.

A conference is being convened, at the instance of Greenwich, of the London borough councils to consider whether it is possible for some authority to maintain a sanatorium for persons suffering from consumption in London, to be supported by a charge upon each of the boroughs. It is considered a general scheme would be more economical than the policy often adopted of maintaining beds in sanatoria in various parts of the country.

The accounts of Leicester Infirmary for the year 1909 show a net balance of income over expenditure of £2,976, which has been applied as follows:—£2,726 to liquidate the deficit existing on January 1, 1909; £250 to reserve fund for contingencies; £13 carried forward to 1910. The reconstruction scheme carried out by Sir Edward Wood, the chairman, has cost £100,000, all of which, with the exception of £850, has been raised.

The annual meeting of the Invalid Children's Aid Association will be held, by kind permisison of her Grace the Duchess of Marlborough, at Sunderland House, Curzon Street, Mayfair, on Wednesday afternoon, April 27, at 3 o'clock. The Duchess of Marlborough will preside, and Robert Hutchison, Esq., M.D., D'Arcy Power, Esq., F.R.C.S., will be among the speakers. Admission by ticket only, application for which may be made to the Secretary, 69 Denison House, Vauxhall Bridge Road, Westminster, S.W.

Anna Maria Helena Comtesse de Noailles, of Hyères, Var, France, and of Eastbourne, whose wills and codicils have recently been the subject of an action in the Probate Division of the High Court, where judgment was pronounced for their validity, has left one-third of her residuary estate to the French Anti-Vivisection Society, to found a sanatorium for nursing young women afflicted with cancer in the breast in its early stage. Admittance to this establishment is to be forbidden to any medical man who is a vivisectionist.

By means of the Samaritan Fund of St. Thomas's Hospital 1,264 patients were sent last year to convalescent homes —an increase of 201 on 1908—and 985 patients were provided with appliances. The reasons for this increase are that one of the long-closed wards was opened and more convalescent treatment was asked for than before. In spite of a legacy of £500, the expenditure exceeded the income by over £20. The patients themselves contributed 18 per cent. of the relief given, and 564 were sent to the Convalescent Home at Swanley built by Mr. Peter Reid.

The new room for the treatment by *x*-rays at the London Hospital has been completed. It is so equipped that it is impossible for the operators to turn on the *x*-ray apparatus until they themselves are outside and beyond the reach of the rays. The Committee reported at the quarterly Court of Governors that the hospitals in East London were considering whether it was possible to obtain better terms in buying provisions and drugs by forming some sort of combination. The matter was still under consideration, but the best arrangement seemed to be that the London Hospital should include the requirements of the smaller hospitals in its own tender, while the contractors would deliver their own goods to the various hospitals of the combination.

